# Assessing vestibular function using electroencephalogram rhythms evoked during the caloric test

**DOI:** 10.3389/fneur.2023.1126214

**Published:** 2023-02-23

**Authors:** Yutong Han, Yanru Bai, Qiang Liu, Yuncheng Zhao, Taisheng Chen, Wei Wang, Guangjian Ni

**Affiliations:** ^1^Academy of Medical Engineering and Translational Medicine, Tianjin University, Tianjin, China; ^2^Tianjin Key Laboratory of Brain Science and Neuroengineering, Tianjin, China; ^3^Key Laboratory of Auditory Speech and Balance Medicine, Tianjin, China; ^4^Institute of Otolaryngology of Tianjin, Tianjin, China; ^5^Key Medical Discipline of Tianjin (Otolaryngology), Tianjin, China; ^6^Department of Biomedical Engineering, College of Precision Instruments and Optoelectronics Engineering, Tianjin University, Tianjin, China

**Keywords:** vestibular function, EEG rhythms, caloric test, nystagmus, cortex

## Abstract

**Introduction:**

The vestibular system is responsible for motion perception and balance preservation in the body. The vestibular function examination is useful for determining the cause of associated symptoms, diagnosis, and therapy of the patients. The associated cerebral cortex processes and integrates information and is the ultimate perceptual site for vestibular-related symptoms. In recent clinical examinations, less consideration has been given to the cortex associated with the vestibular system. As a result, it is crucial to increase focus on the expression of the cortical level while evaluating vestibular function. From the viewpoint of neuroelectrophysiology, electroencephalograms (EEG) can enhance the assessments of vestibular function at the cortex level.

**Methods:**

This study recorded nystagmus and EEG data throughout the caloric test. Four phases were considered according to the vestibular activation status: before activation, activation, fixation suppression, and recovery. In different phases, the distribution and changes of the relative power of the EEG rhythms (delta, theta, alpha, and beta) were analyzed, and the correlation between EEG characteristics and nystagmus was also investigated.

**Results:**

The results showed that, when the vestibule was activated, the alpha power of the occipital region increased, and the beta power of the central and top regions and the occipital region on the left decreased. The changes in the alpha and beta rhythms significantly correlate with nystagmus values in left warm stimulation.

**Discussion:**

Our findings offer a fresh perspective on cortical electrophysiology for the assessment of vestibular function by demonstrating that the relative power change in EEG rhythms can be used to assess vestibular function.

## 1. Introduction

The vestibular system is vital in perceiving spatial position and maintaining balance (1, 2). Damage in the vestibular system would lead to clinical symptoms, such as vertigo (3, 4), and may also affect motor coordination (5, 6) and higher cognitive function (7). A timely and accurate examination of the physiological function of the vestibular system can serve as a crucial guide for determining the cause of associated symptoms in patients as well as for future diagnosis and therapy. The current widely used clinical vestibular function examinations usually include calibration, spontaneous and gaze-evoked nystagmus, the saccade test, smooth pursuit tracking, positional and positioning nystagmus, and caloric test (8). Notably, existing examinations mainly focus on the nystagmus response of the vestibulo-ocular reflex and the balance function of the vestibulo-spinal reflex system (9). One of the main neural circuits in the vestibular system is the vestibulocortical pathway. The thalamic integration center receives information from the vestibular nerve in the vestibulocortical pathway, which then projects it to the vestibular cortex (10). The final stage in the formation of vestibular-related perception is the vestibular-related brain, which is responsible for processing and integrating information. Therefore, changes in neurological function at the cortex may result from or may be the cause of vestibular hypofunction (11). In current clinical examinations, less attention has been paid to the cortex related to the vestibular system. Even though some examinations concern the vestibulocortical pathway, they are not performed directly from the cortical expression but through evaluating various eye movement functions. Therefore, it is of great interest to add a reference to the expression of the cortex level for assessing vestibular functions.

Electroencephalogram (EEG) and sensory-evoked potentials contain abundant physiological and pathological information, which can reflect the excited or inhibited state of the cortex (12). Moreover, EEG technology has the advantages of non-invasiveness, low cost, and high time resolution (13–15). As EEG has been used to diagnose and treat many neurological-related diseases, such as epilepsy and encephalitis (16, 17), it has the potential to provide a neuro-electrophysiological supplement for vestibular function assessment at the cortex level. Moreover, convenient and fast saline electrodes have evolved recently, avoiding the time-consuming and hair-washing problems of using conductive paste.

Existing EEG studies on the vestibular system are mainly aimed at patients with a specific vestibular-related disease or symptoms to explore changes in EEG characteristics. For example, in patients with chronic balance disturbance, alpha rhythm activity in the posterior cingulate cortex rose significantly, while beta activity in several brain regions decreased (18). The severity of symptoms in patients with motion sickness is related to an increase in the gravitational frequency of the power spectral density of the theta rhythm (19). However, vestibular-related diseases and symptoms are diverse and complex (20–22), and there may be other influencing factors other than vestibular hypofunction. Therefore, there is currently a lack of assessments directly targeting vestibular function at the cortex level.

The following two categories of relevant studies are currently available: resting state and evoked state. The EEG microstates in this study were classified into four categories (A, B, C, and D) as in most resting state studies, and the results showed that patients with recurrent otogenic vertigo have a longer duration of the A state and an increased probability of transitioning from A state to D state compared to healthy people (23). The primary triggers for the evoked state are auditory and visual. For example, in the study of auditory-evoked potential, it was found that latencies of wave I peak, wave III–V inter-peaks, Pa, and P300 in patients with vertigo were prolonged (24–27). Moreover, visual-evoked studies found that patients with vestibular migraine had a higher light actuation (28). However, these stimuli use the intimate connection between the auditory and visual pathways and the vestibular route rather than directly activating the vestibular system (29). The vestibular system cannot be directly activated when a non-vestibular evoked stimulation technique is employed, making the evaluation of vestibular function challenging. The horizontal semicircular canal is excited during the caloric test, which is acknowledged as an assessment technique to stimulate and evaluate the vestibular response (30). The caloric test activates the vestibular system directly and is the gold standard for assessing vestibular hypofunction (9).

In this study, we aimed to explore the characteristics of EEG rhythms in different vestibular activation states in the caloric test and to find the characteristics of EEG rhythms for assessing vestibular function. We examined relative power distribution and changes in each EEG rhythm under various vestibular activation states and searched for a relationship between these changes and the caloric test findings. It was found that the alpha power was enhanced during vestibular activation in channels selected *via* significant changes between phases (*p* < 0.05) dominated by the occipital region, and the beta power was reduced in channels selected by the same criteria mainly in the central, top, and occipital regions on the left. It is interesting to note that both changes significantly correlate with nystagmus values in the left warm condition.

## 2. Materials and methods

### 2.1. Subjects

A total of 18 healthy subjects (8 men and 10 women, 23.44 ± 2.33 years) were included in this study. All participants complied with the following requirements: no history of seizures, cardiovascular disease, hypertension, severe head and neck illnesses, ingestion of alcohol within the previous 48 h, ingestion of central excitatory or inhibitory drugs, damage to the external auditory canal or tympanic membrane, or otitis media. The study was approved by the medical ethics review committee at Tianjin University. All subjects gave written informed consent and received payment for participation.

### 2.2. Stimuli in the caloric test

**Figure 1:**
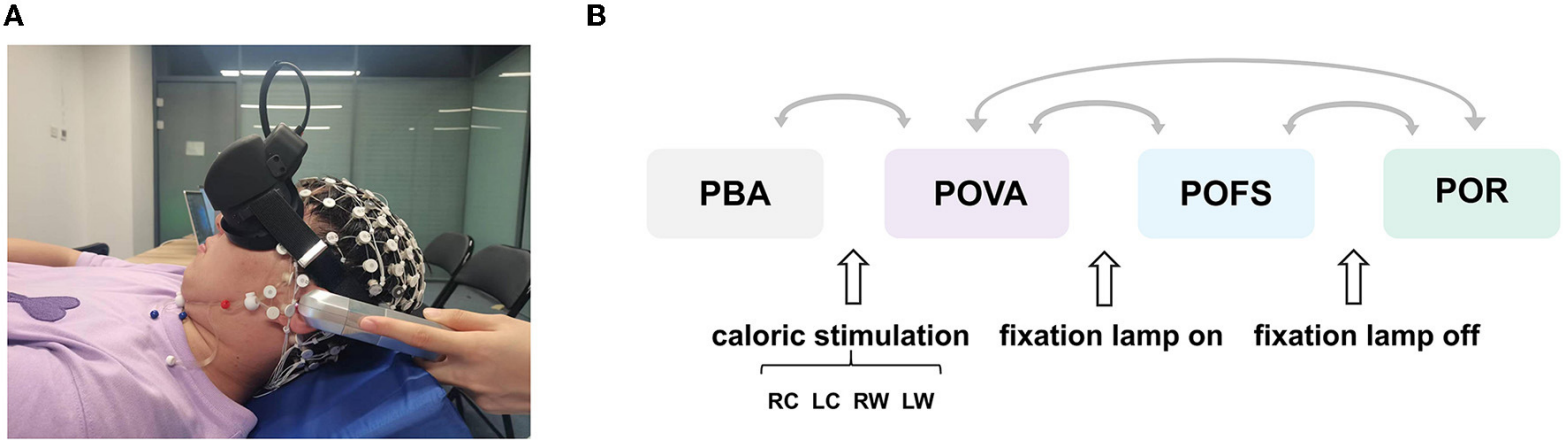
Schematic diagram of the experiment. **(A)** Experimental setup. **(B)** Schematic diagram of the overall experimental flow. The two phases connected by each arrow were compared. PBA refers to the phase before activation, POVA refers to the phase of vestibular activation, POFS refers to the phase of fixation suppression, and POR refers to the phase of removing fixation suppression, respectively. RC, LC, RW, and LW are the abbreviations of right cool, left cool, right warm, and left warm, respectively.

As illustrated in Figure 1A, volunteers were asked to remain awake with their eyes open during the experiment while lying supine in a dark field with their heads raised by 30° on a firm cushion to place their horizontal semicircular canal in a vertical posture. The caloric test was performed by perfusing each ear with cold (24°C) and warm (50°C) gases. Gas perfusion was performed from the external auditory canal in four conditions: cold gas into the right ear (RC), cold gas into the left ear (LC), warm gas into the right ear (RW), and warm gas into the left ear (LW). The duration of each perfusion was 60 s. It is important to ensure that the nystagmus of the subject due to the previous perfusion has completely subsided before each gas perfusion (no less than 5 min). After reaching the maximum nystagmus, the fixation lamp was turned on to suppress the vestibular activation. For each condition, we divided the entire experiment into four phases: the phase before stimulation (PBA), the phase of vestibular activation (POVA), the phase of fixation suppression (POFS), and the phase of recovery (POR), as shown in Figure 1B.

### 2.3. Data recording

During the caloric test, EEG and nystagmus data were concurrently captured.

#### 2.3.1. Nystagmus recording

Before conducting the experiment, calibration was performed. The purpose of calibration is to measure the relationship between the eye movement of a certain angle of view and the corresponding recorded signal (i.e., the displacement of the eye movement curve), which is used to calculate parameters such as amplitude and speed of the eye movement. Then, using video nystagmus (VNG), the maximum slow-phase velocity of spontaneous and caloric test-induced nystagmus from both experiments was captured.

#### 2.3.2. EEG recording

Saline electrode caps (EGI, Geodesic Sensor Net, 64 channels, 1,000 Hz) were used to record EEG data throughout the caloric test, amplified by a Net Amps 400 amplifier. The distribution of electrodes was based on the international 10–20 system, and the impedance was guaranteed to be less than 50 kΩ during the experiment. The volunteers were reminded before the experiment to refrain from unnecessary head and body movements while collecting data to prevent significant data contamination. EEG data for PBA was defined as the data from 50 s before stimulation, for POVA as the data from 20 s centered on the time point of the strongest nystagmus, for POFS as the data from 10 s following the turn-on of the fixation lamp, and for POR as the data from 10 s following the turn-off of the fixation lamp.

### 2.4. EEG data analysis

#### 2.4.1. Preprocessing

Raw EEG data were preprocessed using EEGLAB, an open-source toolbox of Matlab (R2021a). To lessen the impact of high-frequency noise and low-frequency interference on the data, 30 Hz low-pass filters and 0.5 Hz high-pass filters were first used. We averaged the reference value of data across all electrodes. The spherical approach was used to interpolate the data of electrodes that were detached from the skin during the experiment. To improve the computational efficiency of subsequent processing and analysis, data were down sampled to 200 Hz. Independent component analysis was performed to eliminate artifacts like a heartbeat, muscle tension, and eye movement.

#### 2.4.2. Power spectral density of EEG rhythms

The Welch method was employed to determine the power spectral density of data. The energy of each EEG signal was normalized to reduce the potential difference among subjects. The relative power of EEG rhythms delta (0.5–4 Hz), theta (4–8 Hz), alpha (8–14 Hz), and beta (14–30 Hz) was calculated for each phase. The topographic maps of each phase were created using the relative power findings of all channels to explore the distribution of certain EEG rhythms. Variations in the global brain distribution of each EEG rhythm were observed and examined between phases.

### 2.5. Statistical analysis

The statistical analysis was carried out using SPSS (26.0). The Wilcoxon signed-rank test was used to examine the relative power findings for each channel for each EEG rhythm across phases shown in Figure 1B, as some of the data did not follow a normal distribution. POVA and PBA, POFS and POVA, POR and POFS, and POR and POVA were contrasted independently to identify the changes caused by caloric vestibular stimulation, fixation suppression, removal of fixation suppression, and following the process of fixation suppression. The channels with significant changes between phases (*p* < 0.05) were selected as the region of interest, and the changing trend of the region of interest was calculated. Additionally, the Pearson correlation test was utilized to determine the discrepancy between the relative power change of the particular EEG rhythm and the result for nystagmus.

## 3. Results

### 3.1. Topographic map of EEG rhythms

**Figure 2:**
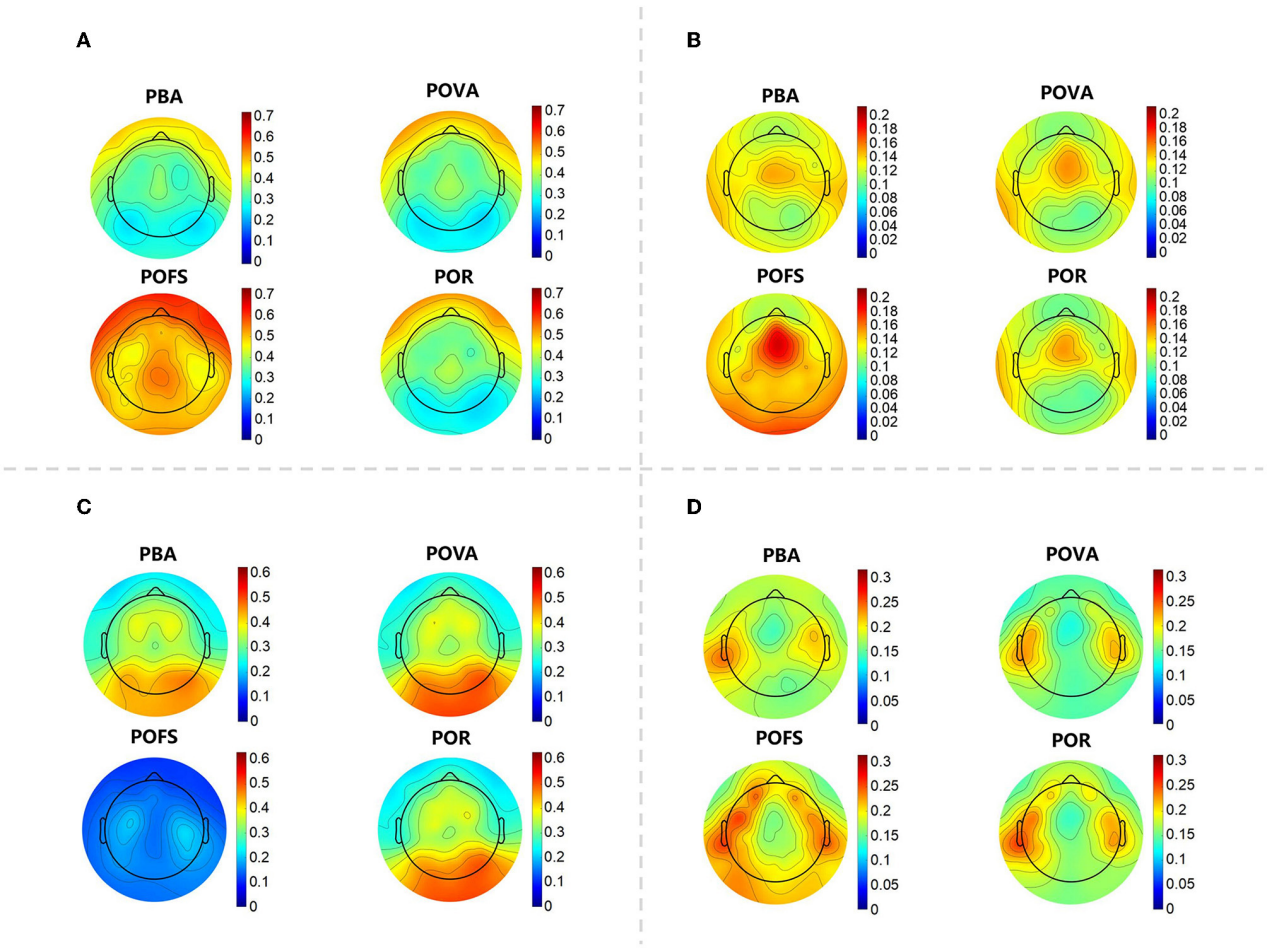
The topographic map of each EEG rhythm in each phase. **(A)** The topographic map of the relative power of the delta rhythm in the four phases. **(B)** The topographic map of the relative power of the theta rhythm in the four phases. **(C)** The topographic map of the relative power of the alpha rhythm in the four phases. **(D)** The topographic map of the relative power of the beta rhythm in the four phases.

The whole-brain delta rhythm was observed to have a relative power distribution that was strongest in the forehead in PBA, slightly enhanced in the forehead in POVA, the whole brain enhanced in POFS, and then returned to its previous state between PBA and POVA in POR, leaning more toward POVA. The center frontal region was comparatively strong in PBA, while the forehead and occipital regions were slightly weaker in POVA, according to the observation of the relative power distribution of the whole-brain theta rhythm. The central frontal and occipital regions were considerably enhanced during POFS; even after enhancement, the central frontal region remained the strongest. While the distribution in POR was comparable to that in POVA, the frontal center was weaker. According to an analysis of relative power distribution in the whole-brain alpha rhythm, the energy of the occipital region was more pronounced in PBA and increased in POVA. In POFS, the entire brain was repressed. The relative alpha power in the occipital region in POR was high, and its intensity was midway between PBA and POVA. The relative power of beta rhythm in the temporal region was relatively strong in PBA and suppressed in POVA. The relative beta power in the frontotemporal and occipital regions was enhanced in POFS, and the enhancement of these regions was weakened in POR (as shown in Figure 2).

### 3.2. Significant differences and trends in EEG rhythm changes among phases

**Figure 3:**
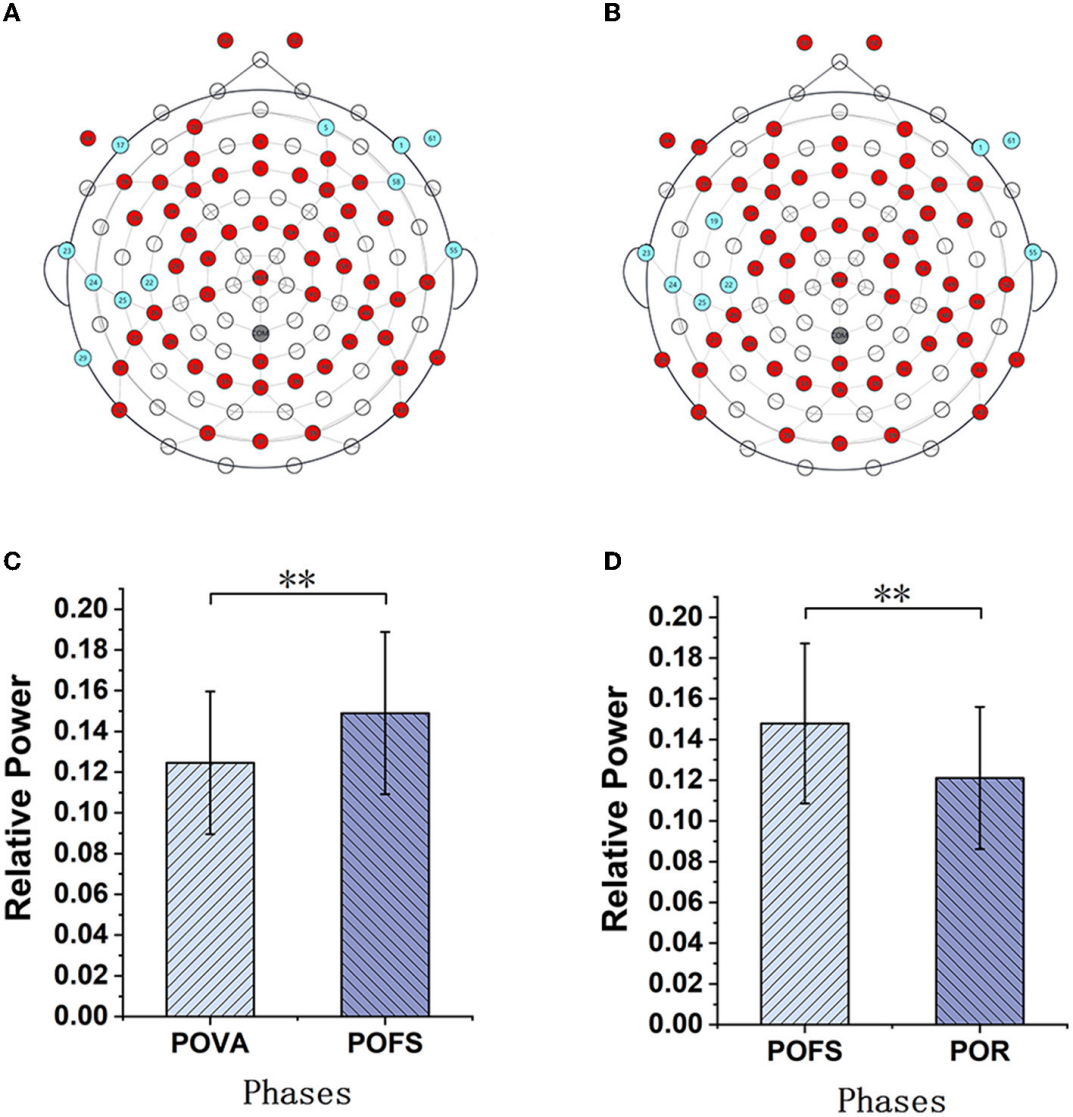
The comparison of theta power in different phases. **(A)** and **(B)** are the brain topography distributions of selected channels in the POVA and POFS comparison and POFS and POR comparison, respectively. In the brain topography, the red dots are channels with a significant difference from the comparison of the two phases, the blue dots are channels without significant differences, and the white dots are empty channels. According to the chosen channels shown in red in **(A, B)**, the average relative power changes for POVA and POFS comparison and POFS and POR comparison, respectively, are shown in **(C, D)**. ^**^p < 0.01.

**Figure 4:**
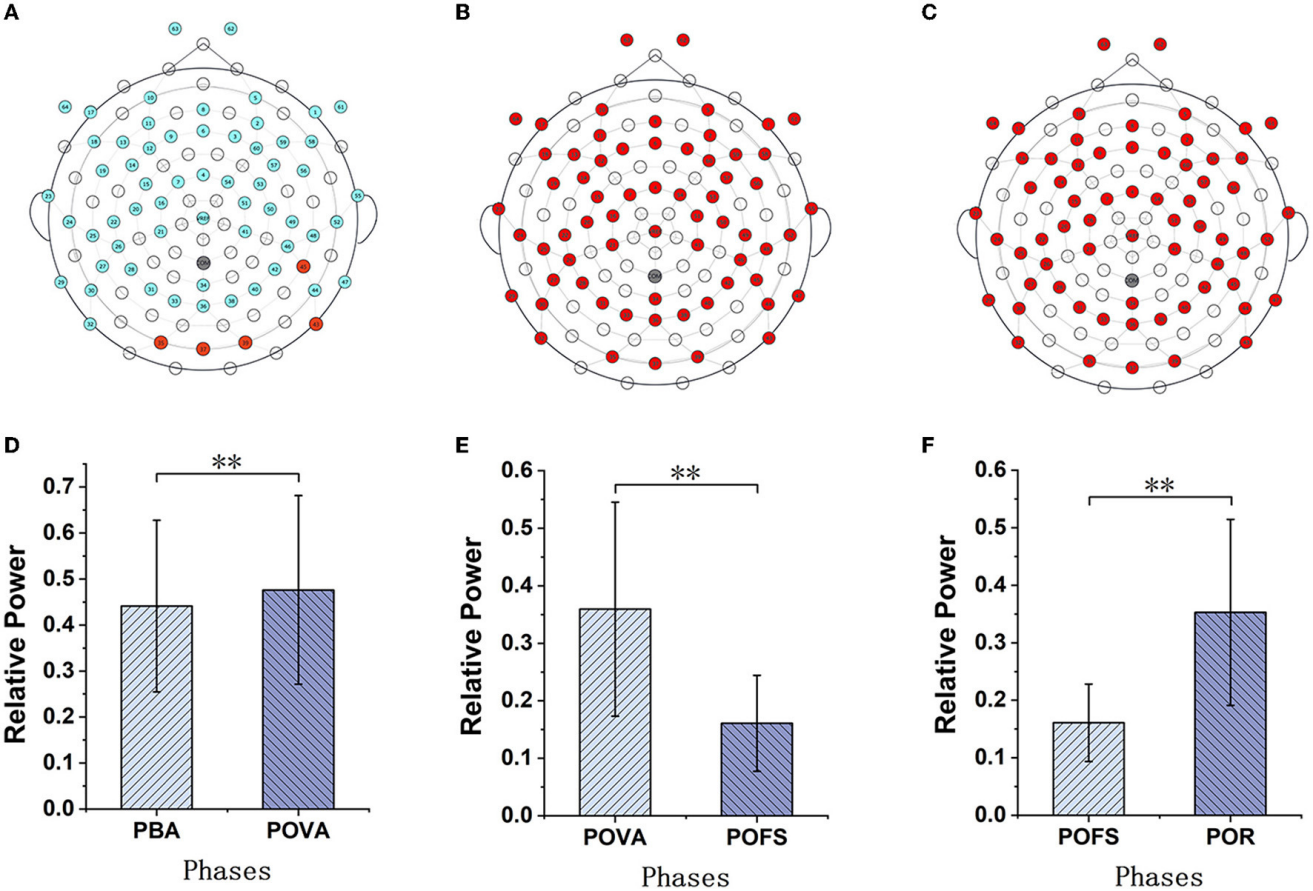
The comparison of alpha power in different phases. **(A–C)** are the brain topography distributions of selected channels in the PBA and POVA comparison, POVA and POFS comparison, and POFS and POR comparison, respectively. In the brain topography, the red dots are channels with significant differences between the two comparisons, the blue dots are channels without significant differences, and the white dots are empty channels. Mean relative power changes according to the selected channels are shown in red in **(A–C)** for the PBA and POVA comparison, the POVA and POFS comparison, and the POFS and POR comparison are shown in **(D–F)**, respectively. ^**^p < 0.01.

**Figure 5:**
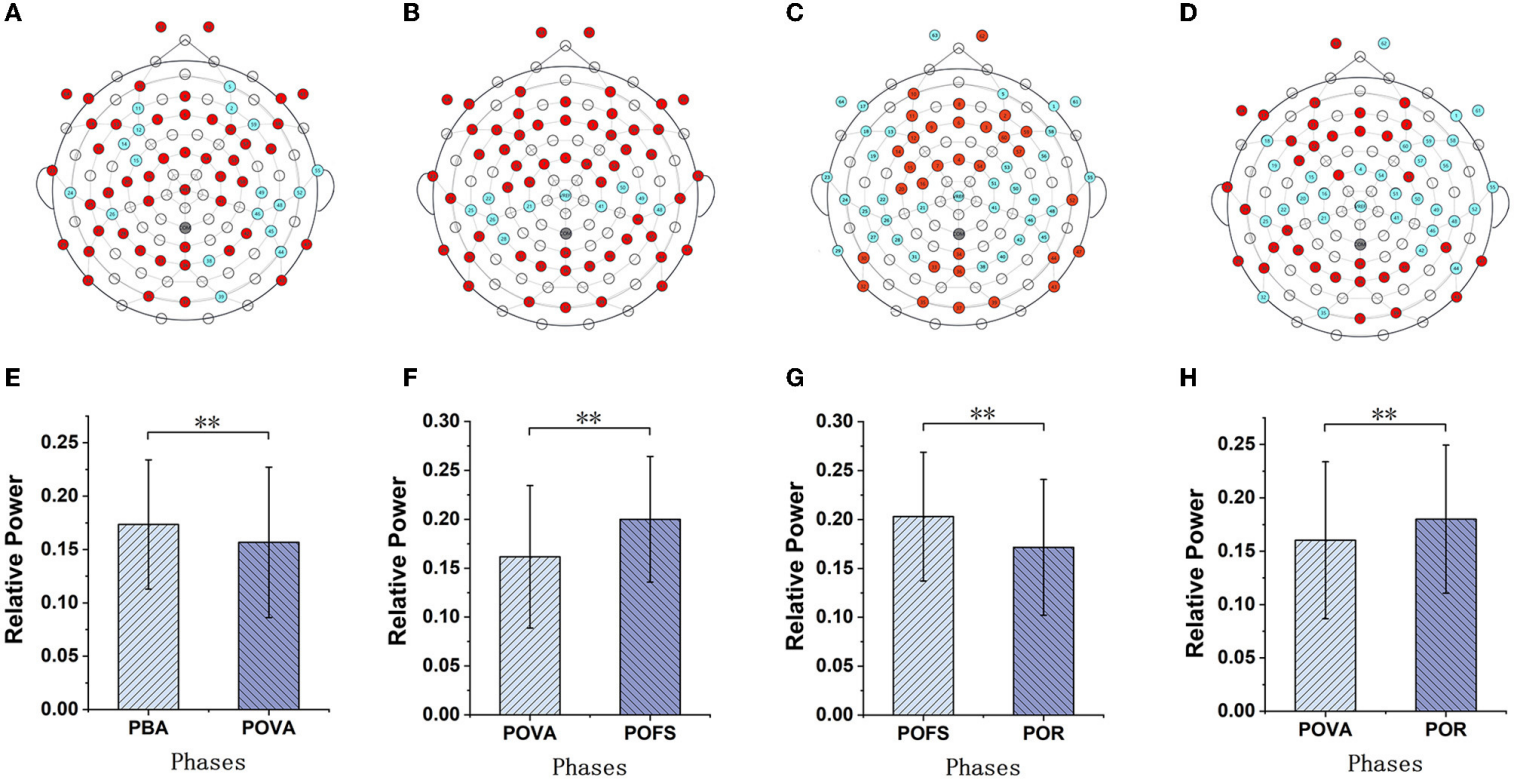
The comparison of beta power in different phases. **(A–D)** are the brain topography distributions of selected channels in PBA and POVA comparison, POVA and POFS comparison, POFS and POR comparison, and POVA and POR comparison, respectively. In the brain topography, the red dots are channels with significant differences between the two comparisons, the blue dots are channels without significant differences, and the white dots are empty channels. Mean relative power changes according to the selected channels shown in red in **(A–D)** for the PBA and POVA comparison, POVA and POFS comparison, POFS and POR comparison, and POVA and POR comparison are shown in **(E–H)**, respectively. ^**^p < 0.01.

Channels with significant differences (*P* < 0.05) were chosen when theta, alpha, and beta rhythms were evaluated in each phase, as illustrated in Figures 3–5. The changing trend of relative power calculated based on the selected channels was also shown in the lower panels of Figures 3–5. It was discovered that the central frontal, parietal, and occipital areas included most channels exhibiting appreciable variations in theta power. The significantly changed region of the alpha rhythm was occipital and slightly right-lateralized in the comparison of PBA and POVA, and the significantly changed region was the whole brain in POVA and POFS comparison and POFS and POR comparison. Comparing PBA and POVA, significant alterations in beta rhythm were mainly in the central, parietal, and left occipital regions. In the comparison between POVA and POFS, significant changes were evident in the whole brain except for the central region. In comparing POFS, POR, and POVA and POR, significant changes in channels were concentrated in the frontal and parieto-occipital regions. Table 1 summarizes the trends in the relative power of EEG rhythms between phases obtained from the chosen channels.

**Table 1:** The trends of the relative power of EEG rhythms in selected channels between phases.

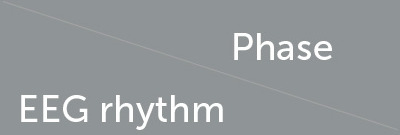	**POVA vs. PBA**	**POFS vs. POVA**	**POR vs. POFS**	**POR vs. POVA**
Theta	/	Increase	Decrease	/
Alpha	Increase	Decrease	Increase	/
Beta	Decrease	Increase	Decrease	Increase

### 3.3. Correlation between relative power of EEG rhythms and nystagmus value

The nystagmus value is an index to evaluate the response to vestibular stimulation in the clinic. The nystagmus results were collected synchronously during the EEG recording in the experiment, as listed in Table 2. The relative strength of the alpha and beta rhythms between the POVA and PBA phases demonstrated a substantial difference in the selected brain regions, according to the results of EEG rhythms in various phases. Therefore, under four settings (RC, LC, RW, and LW), a correlation study was done between the change in the relative power of the alpha/beta rhythm and the nystagmus value.

**Table 2:** Spontaneous and caloric-stimulated nystagmus values.

**Subject number**	**Spontaneous nystagmus**	**Right warm**	**Left warm**	**Left cold**	**Right cold**
1	R1.6	R9.6	L8.7	R5.6	L3.0
2	R0.6	R9.4	L12.8	R10.5	L16.2
3	R2.7	R7.5	L5.3	R4.0	L9.5
4	R1.2	R4.8	L7.3	R8.0	L4.1
5	L0.9	R3.4	L10.3	R4.1	L7.1
6	L1.2	R5.0	L4.5	R3.7	L7.1
7	L0.5	R3.2	L3.2	R6.6	L7.0
8	R1.0	R7.6	L3.8	R18.8	L11.0
9	L0.7	R0.5	L3.1	R2.2	L3.9
10	L0.2	R12.1	L5.4	R4.2	L8.7
11	L0.5	R4.0	L1.3	R4.7	L7.5
12	L1.7	R1.1	L5.0	R1.8	L6.6
13	R0.6	R4.9	L1.4	R5.1	L3.2
14	L0.3	R2.8	L2.0	R1.2	L2.3
15	L1.3	R2.7	L5.5	R9.0	L7.1
16	R1.3	R4.9	L17.1	R7.7	L6.2
17	0	R8.9	L9.7	R11.0	L10.8
18	0	R5.4	L14.7	R8.7	L10.9

L and R refer to the direction of nystagmus as left and right, respectively.

**Figure 6:**
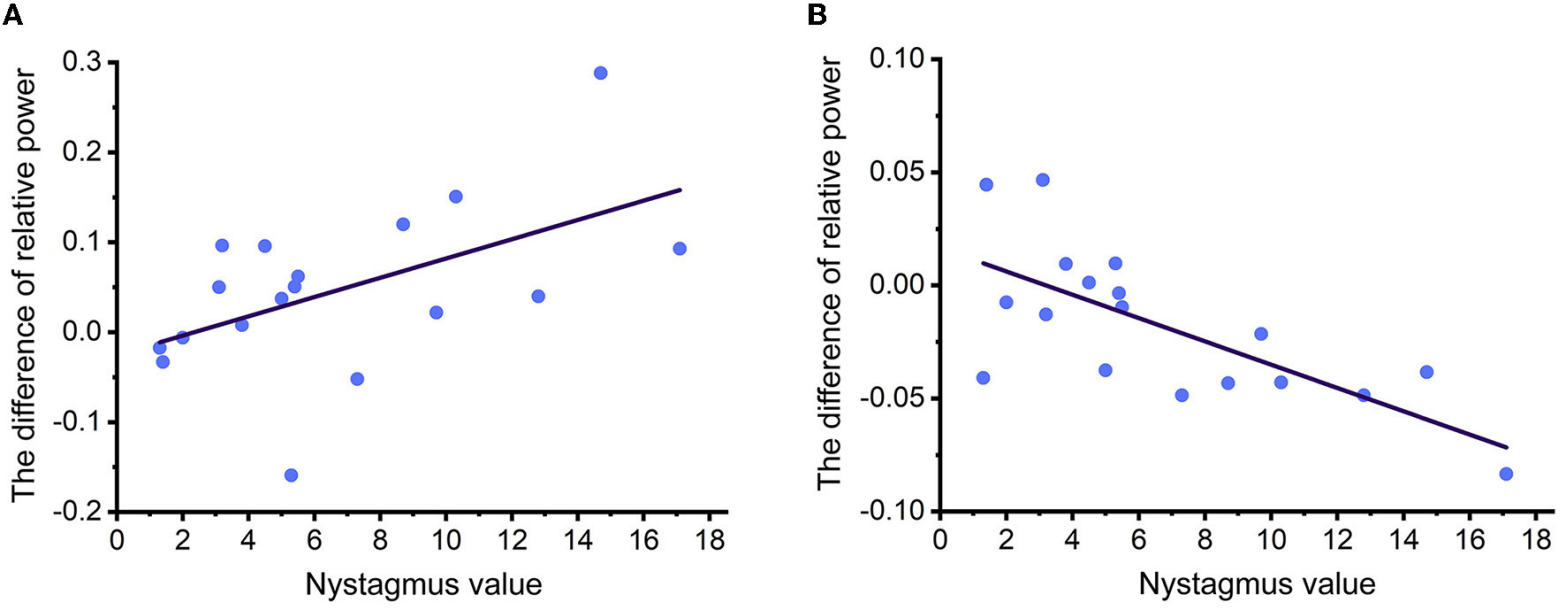
**(A, B)** The fitting curve of correlation coefficients between relative power of EEG rhythms and nystagmus values under LW condition.

In the four conditions, a significant correlation between the relative power of EEG rhythms and nystagmus values was observed in the LW condition for both alpha and beta rhythms, as shown in Figure 6. It was discovered that the enhancement of alpha power in the region of interest was significantly correlated with the nystagmus value (r = −0.527, *p* = 0.025), and the reduction of beta power in the region of interest was also significantly correlated with the nystagmus value (r = 0.708, *p* = 0.001).

## 4. Discussion

This study aimed to examine how the brain reacts to vestibular stimulation and evaluate vestibular function from a cortical electrophysiological standpoint. A caloric test was used to provide direct vestibular stimulation, and brain responses were compared among the following four phases: PBA, POVA, POFS, and POR. The results revealed that the changes in EEG cortex expression under different vestibular activation conditions were significantly different. Notably, in the case of LW stimulation, the enhancement of alpha power and the decrease in beta power in specific brain regions were related to nystagmus values. Therefore, the study offers a novel perspective for assessing vestibular function using characteristic EEG rhythms.

### 4.1. The basis for the division of phases in the caloric test

The semicircular canals in the peripheral vestibular receptors sense rotational angular acceleration stimulation. Caloric stimulation is an examination that uses caloric vestibular stimulation to stimulate the horizontal semicircular canals to induce and observe vestibular responses (31). When the external ear canal is exposed to cold or warm stimuli, the temperature alteration affects the outer semicircular canal *via* the tympanic membrane, the tympanic chamber, and the bone wall. In the outer semicircular canal, the specific gravity of the endolymph fluid changes due to thermal expansion and contraction, resulting in the convection phenomenon of “warm rises and cool drops” of the endolymph fluid (32). Nystagmus can be seen as a symbol of vestibular stimulation response. The fixation lamp on denotes the start of central inhibition, and the fixation lamp off signals the removal of central inhibition. These critical timings divide the caloric test into different states of vestibular activation. Therefore, although EEG was recorded throughout the entire caloric test, EEG data were divided into four phases for analysis, corresponding to PBA, POVA, POFS, and POR, respectively.

### 4.2. Beta power has the potential to be used for assessing vestibular function

Regular changes in beta rhythm activity were found in the caloric test, in accordance with the observation made in a study by Brkić about EEG abnormalities in patients with central vertigo utilizing vestibular caloric stimulation. Compared to the control group, more patients showed changes in beta activity during warm stimulation (33). Consistent conclusions indicated that the changes in beta rhythm in this study were induced by caloric vestibular stimulation; in addition, we carried out a quantitative analysis comparing the conclusion of this study with those of the work of Brkić. Brkić did not observe significant changes in healthy subjects and therefore suggested that damage to specific thalamocortical connections was responsible for the EEG changes. In contrast, we found changes in beta rhythm in healthy individuals. We speculate that this may be due to various experimental factors, such as temperature, duration of stimuli, and potential equipment limitations.

We discovered that the relative power of the beta rhythm significantly decreased during the vestibular stimulation response phase. Many classical observations have linked this rhythm to motor function (34). Beta-rhythm activity may have a role in sensorimotor integration. Many authors argue that decreased beta power reflection indicates the activation of the sensorimotor associated with an increase in corticospinal excitability (35), which may correspond to the sensations of dizziness and rotation during the vestibular stimulus response phase, indicating that subjects may be involved in motion perception processes during vestibular stimulus activation. The outcome supports the hypothesis that beta rhythm is strongly related to information processing in the sensory-motor systems. The relative power of the beta rhythm in the central inhibition phase significantly increased and was found to be higher than before, demonstrating that the cerebral cortex response was suppressed. The result was consistent with the observation in the positron emission computed tomography (PET) study of caloric stimulation, in which Naito found that the vestibular response was inactivated during visual fixation (36). After the inhibition was canceled, the beta power decreased again and returned to the same level as the phase before stimulation, indicating that the vestibular stimulation response was no longer suppressed.

The regulation of changes in beta power found in this study corresponds to the activation and inhibition phases in the caloric test. We suggest that beta rhythm is closely related to the state of vestibular activation, and the degree of beta power suppression, especially in LW stimulation, correlates with the intensity of nystagmus. Therefore, it is reasonable to conclude that the relative power of beta rhythm caused by caloric vestibular stimulation reflects the vestibular function condition.

### 4.3. Enhanced alpha power in the occipital region may be associated with vestibular activation

In a PET study, the unilateral caloric test was used to provide vestibular stimulation to healthy subjects, and it was found that the cortical activation pattern of healthy people during vestibular stimulation was identical to that in patients with vestibular neuritis (37–40). According to an EEG study that compared patients with chronic vestibular symptoms to healthy individuals, the occipital region exhibited a difference in alpha activation (18), which was consistent with the change in the region of alpha rhythm that our study found. While these previous studies support the inference that vestibular stimulation does elicit alpha power changes in this study. Considering that alpha waves usually play an important role in regulating cognition and attention (41, 42) and can also inhibit task-irrelevant neural representations (43), it is speculated that this enhanced alpha rhythm caused by vestibular stimulation may be related to the inhibitory mechanism of attentional disengagement. We found that the enhancement of alpha power was also associated with the degree of nystagmus during vestibular stimulation. Therefore, the degree of alpha power enhancement could be used to evaluate the degree of vestibular activation in clinical practice.

### 4.4. The necessity of dark field in experimental design

According to some studies, in the closed-eye state, or when the individuals kept their eyes open and were alert in the dark field, alpha power was higher than that in the open field (44–46). We used electronystagmography with an eyecup in the caloric test to create darkroom conditions. In other words, the dark field applied in the caloric test might result in different results for the relative power of EEG rhythms due to the change in the proportion of alpha rhythm. To this end, we performed a comparative experiment by performing the caloric test with an eyecup hood opening. Unfortunately, the results revealed that subjects had neither noticeable nystagmus nor obvious EEG rhythm changes during vestibular stimulation. It is speculated that the condition of open view has a similar effect on central inhibition as turning on the fixation lamp. Therefore, the caloric test can only be carried out in a dark field to achieve vestibular induction. Since all data in our experiments were collected in the dark field, we believe that the change in alpha power is associated with vestibular activation.

### 4.5. Clinical significance and future directions

Some previous studies have attempted to classify patients and healthy individuals based on resting-state EEG characteristics (18) or auditory-evoked potential characteristics (24–27). Brain responses to vestibular stimulation have been observed (47), but less research has been conducted to explore the possibility of EEG characteristics for assessing vestibular function, as mentioned in this study. The EEG characteristics induced by vestibular stimulation in healthy controls reflect vestibular-evoking conditions similar to those found in patients with vertigo while avoiding the influence of other non-vestibular causes of vertigo. Therefore, this study is of great significance for understanding the mechanisms of vestibular-evoked brain responses and will help to distinguish patients with vertigo from healthy controls more accurately in the next step.

Video nystagmus is widely used in the clinical examination of vestibular dysfunction, but some patients, especially elderly patients, have difficulty keeping their eyes open required by the nystagmus test during the caloric test, resulting in inaccurate results. The EEG responses to vestibular stimulation are not limited by this, increasing its clinical utility. We conclude that EEG characteristics can be used as complementary indicators to evaluate vestibular function in clinical applications in the future.

## 5. Conclusion

To overcome the lack of specific biomarkers at the cortex level in the current vestibular function assessment, we combined EEG technology and the caloric test to compare the characteristics of EEG rhythms in different phases of the caloric test. We investigated the correlation between changes in EEG rhythm and nystagmus indicators. The results indicate that the inhibition of beta power in the central top and left occipital regions and the enhancement of alpha power in the occipital region in the EEG rhythm can be taken as the cortical electrophysiological characteristics of the response to caloric vestibular stimulation. The changes in the relative power of alpha and beta rhythms in designated brain regions are significantly related to the intensity of nystagmus induced by caloric vestibular stimulation, which verifies the effectiveness of the selected EEG characteristics to be used for assessing vestibular function.

## Data availability statement

The raw data supporting the conclusions of this article will be made available by the authors, without undue reservation.

## Ethics statement

The studies involving human participants were reviewed and approved by Ethics Review Committee of Tianjin University. The patients/participants provided their written informed consent to participate in this study.

## Author contributions

GN, YB, TC, and WW contributed to the conception and design of the work. YH and QL collected data. YH and YZ analyzed data. GN, YB, and YH drafted and revised the manuscript. All authors contributed to the article and approved the submitted version.
